# Odor-Active Compound Stability in Mango Peel Side-Streams: Insights for Valorization and Waste Minimization

**DOI:** 10.3390/foods15020215

**Published:** 2026-01-08

**Authors:** Rodrigo Oliver-Simancas, María Consuelo Díaz-Maroto, Álvaro Fernández-Ochoa, María Soledad Pérez-Coello, María Elena Alañón

**Affiliations:** 1 Division of Industrial Biotechnology, Department of Life Sciences, Chalmers University of Technology, 41296 Gothenburg, Sweden; simancas@chalmers.se; 2 Department of Analytical Chemistry and Food Science and Technology, Faculty of Chemical Sciences and Technologies, University of Castilla-La Mancha, Avda. Camilo José Cela 10, 13071 Ciudad Real, Spain; mariaconsuelo.diaz@uclm.es (M.C.D.-M.); soledad.perez@uclm.es (M.S.P.-C.); 3 Regional Institute for Applied Scientific Research (IRICA), Area of Food Science and Technology, University of Castilla-La Mancha, Avda. Camilo José Cela 10, 13071 Ciudad Real, Spain; 4 Department of Analytical Chemistry, Faculty of Sciences, University of Granada, Campus Fuentenueva, 18071 Granada, Spain; alvaroferochoa@ugr.es; 5 Department of Analytical Chemistry and Food Science and Technology, Higher Technical School of Agronomic Engineering, University of Castilla-La Mancha, Ronda de Calatrava 7, 13071 Ciudad Real, Spain

**Keywords:** mango side-streams, revalorization, volatile compounds, odor-active compounds, ripening, maturity, thermal processing

## Abstract

Comprehensive characterization of the mango peel volatilome is essential to revealing its aromatic potential and enabling its revalorization as a natural flavoring. The volatile profile of *Mangifera indica* L. var. Osteen peels at three ripening stages (green, ripe, overripe) was analyzed before and after thermal drying (45 °C, 18 h): an unavoidable stabilization step for valorization applications. HS–SPME/GC–MS enabled the identification of 76 volatile compounds across different key aroma-contributing families: monoterpenes, sesquiterpenes, alcohols, aldehydes, ketones, esters, furanics and norisoprenoids. The ripening stage significantly influenced the qualitative and quantitative volatilome in fresh samples but drying heavily reduced those differences. Multivariate analyses confirmed that the drying process is the dominant factor shaping the stabilized peels’ volatilome. These findings underscore the industrial relevance of this side-stream: regardless of ripening stage, mango peels can be uniformly stabilized to be upcycled into aroma-rich ingredients. It simplifies raw material sourcing and supports food waste revalorization strategies in flavor and fragrance developments.

## 1. Introduction

Mango (*Mangifera indica* L.) is one of the three most significantly traded tropical fruits worldwide, according to FAO (2024) data for 2023 [1]. The Food and Agriculture Organization of the United Nations (FAO) considers mango, mangosteen and guava in the same commodity cluster, which accounted for approximately one-quarter of global major tropical fruit trade in both quantity and constant value terms in 2023 (2.3 million tonnes and 1173 USD per ton, respectively) [1].

Renowned for its exquisite and distinctive flavor, mango has ranked among the most valued fruits globally in recent decades, as evidenced by a trend in production and trading data [1]. Its flavor is often described as a blend of citrus, peach and pineapple with a sweet and tangy taste [2]. Although mango is mainly traded as fresh fruit, its versatility and rich culinary traditions have greatly boosted the demand for processed mango products in recent years [1].

Mango processing generates significant amounts of non-edible waste (mainly peel and seed), which can account for 35–55% of the fruit’s total mass [3]. Although these significant by-products are usually discarded, new revalorization strategies have emerged in recent years, aiming to create added-value products from previously underutilized materials. Most of these efforts have been focused on extracting specific chemical compounds (bioactive compounds, antioxidants, natural flavorings…) from these discarded parts to develop functional ingredients, nutraceuticals or eco-friendly alternatives to synthetic additives.

Over the last decade, some studies have been conducted to elucidate the chemical composition of mango peel with an eye to its harness and potential applications in nutraceuticals and food industries [4,5,6,7]. Particularly, mango peel, which can entail up to 20% of the fruit is total weight [3] seems to contain a significant concentration of volatile compounds, even more abundant than in the edible flesh [3,8]. These volatile compounds, responsible for the characteristic mango flavor, include a wide variety of substances such as monoterpenes, sesquiterpenes, aldehydes, alcohols, norisoprenoids, furans and esters, among other compounds [8,9,10]. Therefore, mango peel contains odor-active compounds that could be leveraged for peel valorization in the food industry as natural flavorings. However, to harness its potential, it is important to consider that the composition and concentrations of aromatic compounds in mangoes are influenced by various factors, including the cultivar and geographical origin [10,11,12,13,14,15], part of the fruit [8,16] or processing [17,18,19], among other factors. Among the factors influencing fruit flavor, the maturity stage is likely the most significant. Fruit flavor is a dynamic process, with volatile substances continuously synthesized and evolving throughout fruit growth and ripening [9,12,15,18,20,21,22].

In this context, mango peel side-streams can be produced at various stages of ripening, depending on the mango-based food product being manufactured. For instance, some of the most popular food items made from mango pulp include chutneys, pickled mango, or amchurs, for which under-ripe mangoes are required [23]. However, in the juice and derived beverages industries, mangoes that are ripe to a higher degree are particularly preferred because they result in higher yields of the liquid fractions. Therefore, the volatile composition of peel by-products generated can vary significantly depending on the originating industry. In addition, the drying process is required for the stabilization of the high-moisture content (around 90%) and enabling the preservation of this by-product until the precise time for its revaluation but can also alter the native volatile composition. Common drying methods used in the food industry include sun-drying, which depends on variable weather conditions, as well as heat-drying and freeze-drying, both of which operate under controlled conditions. Comparing various drying methods, mango peels dried at lower temperatures (45 °C for 18 h) exhibited significantly lower losses of volatile compounds compared to those dried at higher temperatures (60 °C for 12 h) or even those subjected to freeze-drying [19]. It has been reported that the concentrations of furanic compounds and some C13-norisoprenoids can be increased by the temperature used in thermal treatments [19]. The increase in furanic compounds seems to be attributable to the different carbohydrate degradation pathways, such as enzymatic reactions, non-enzymatic browning or simply by a gentle heating of the existing sugars of mango peels [24]. Meanwhile, the increase in C13-norisoprenoids is due to the enzymatic hydrolysis of their glycosylated forms, as a consequence of the heat and time conditions applied during the thermal method [25].

Despite the recognized importance of the ripening stage and drying in shaping the aromatic profile of these vegetable matrices, no previous studies have systematically examined how these two factors interact to influence the volatile composition of mango peel by-products. Therefore, this study aims to investigate how the ripening stage of mango fruit and the post-harvesting process of convective drying affect the volatile composition of mango peel. To this end, a comprehensive volatilome analysis was conducted, aiming to investigate the impact of these factors on the aromatic profile of stabilized mango peels. Based on univariate and multivariate statistical analyses, the most favorable valorization and sustainable management towards scent-related applications were studied.

## 2. Materials and Methods

### 2.1. Mango Peels and Sample Preparation

Approximately thirty Osteen mangoes cultivated in the Spanish Tropical Coast were provided by Miguel García Sánchez e Hijos S.A. (Motril, Spain) at three different ripening stages, categorized based on their °Brix levels as follows: green (8.3 ± 1.5 °Brix), ripe (15.1 ± 1.1 °Brix) and overripe (20.8 ± 0.4 °Brix). Mangoes were washed thoroughly in warm tap water to eliminate any potential dust or dirt. A manual peeler was used to remove the mango peels, and the peels were kept in a sealed plastic bag at a temperature of 4 °C to prevent modifications to the volatile profile until the moment of analysis or following treatments. Each batch of peels at a different maturation stage was homogenized and divided into two groups: one of them was to be directly analyzed, the fresh samples, and the other group was to be submitted to a drying process, the dried samples. Fresh samples, with a moisture content ranging between 74.25 and 78.16% (Table 1), were cut into 1 × 1 mm pieces and kept at −80 °C until their analysis or treatment. At the time of volatilome analysis, the samples were defrosted for three hours in refrigeration conditions at 4 °C. Subsequently, the volatile profile analysis was conducted immediately. The samples were categorized as follows: Fresh Green (FG), Fresh Ripe (FR) and Fresh Overripe (FO). On the other hand, for obtaining the dried peels, the samples were also defrosted at 4 °C for three hours and immediately stabilized to decrease their water content below 8% of relative humidity, ensuring a secure moisture value from a microbiological and enzymatic point of view. This step was carried out following a method optimized by Oliver-Simancas et al., 2020 [19], which consisted of oven drying with natural convection at 45 °C for 18 h (J.P. Selecta, 2000237, Barcelona, Spain). In this case, samples were coded as follows: dried green (DG), dried ripe (DR) and dried overripe (DO). The relative humidity of dried samples ranged between 7.89 and 8.20% (Table 1) and was determined in triplicate by an adapted international standard method (ISO 1442:2023) [26] to allow for the normalization of volatile compound concentrations on a dry matter basis. Finally, the dried samples were ground with an electric mill and sieved through a 0.7 mm mesh to obtain a homogeneous powder prior to analysis.

### 2.2. Analysis of Volatile Profile from Mango Peel by HS–SPME/GC–MS

Volatile compounds were analyzed by HS–SPME/GC–MS according to a previously described method [19]. Fresh (2 g) and dried (0.5 g) mango peel samples were placed in sealed vials with Milli-Q water, NaCl and 4-nonanol as an internal standard. Different sample amounts were used to compensate for concentration effects associated with drying. Volatile extraction was performed using a DVB/CAR/PDMS fiber (Supelco Co., Bellefonte, PA, USA) under controlled temperature and agitation conditions. After thermal desorption in the GC injector, compounds were analyzed in splitless mode. Separation was achieved on a polar DB-WAX column, and mass spectra were acquired by electron impact ionization (70 eV) over a scan range of 45–550 *m*/*z*.

Compound identification was performed using authentic standards when available; otherwise, tentative identification was based on linear retention indices and mass spectra matching against commercial libraries and literature data (similarity indices > 90%). Quantification was performed by means of internal standard calibration curves previously calculated and reported in the literature [19] using the following formula:

$$Concentration\;Target\;Compound=\frac{Peak\;Area\;Target\;Compound}{Peak\;Area\;Internal\;Standard}\;\times Concentration\;Internal\;Standard$$

Each sample was analyzed in triplicate and results were expressed in micrograms per gram of dry extract matter (µg/gDM) except for furanic and C13-norisoprenoids compounds, which were expressed in nanograms per gram of dry matter (ng/gDM).

### 2.3. Sensory Evaluation

The evaluation was conducted in a standard sensory analysis chamber, adhering to the ISO 8589:2007 guidelines [27]. Sensory panel was composed of eight trained and experienced judges operating in accordance with ISO 8586 [28]. Prior to the evaluation, the final set of descriptors was defined through a brief familiarization session with mango peels, during which literature-derived aroma descriptors [13,29] were reviewed and agreed upon following a preliminary smelling round until consensus was reached. Samples were blind-sniffed by the judges (five women and three men, aged 24–62 years). For the olfactory assessment, samples were presented in methacrylate olfaction domes with a manual opening–closing system. Panelists used a 10 cm unstructured scale to rate descriptor intensity, where the left end indicated the absence of the attribute and the right end its maximum perceived value. Each evaluation was performed in triplicate across three different weeks to ensure repeatability and minimize day-to-day variability.

### 2.4. Statistical Analysis

Statistical analysis was conducted using the IBM SPSS Statistics v. 24.0 for Windows statistical package. Each experimental condition was analyzed in triplicate (*n* = 3). The volatile data set was subjected to the Student–Newman–Keuls test to identify significant differences between the samples. For multivariate analysis, the dataset was log10-transformed and auto-scaled (mean-centered and divided by standard deviation) to ensure comparability among variables with different magnitudes. MetaboAnalyst 5.0 (www.metaboanalyst.ca), a free web-based software focused on data processing and analysis, was used to generate a normalized hierarchical clustered heatmap and principal component analysis (PCA) with corresponding loading plots. Euclidean distance was selected as the similarity measure and Ward’s linkage as the clustering algorithm. A *p*-value threshold of <0.05 was used to determine statistical significance in both univariate and multivariate analyses.

Sensory data were analyzed by analysis of variance (ANOVA) using IBM SPSS Statistics for Windows (version 24.0) to assess the effects of the ripening stage and drying treatment on the evaluated sensory attributes. Significant differences among samples were determined at *p* < 0.05, followed by a Student–Newman–Keuls post hoc test and multiple comparison tests, when appropriate, to identify significant differences among samples.

## 3. Results and Discussion

### 3.1. Relative Humidity Content of Samples

As shown in Table 1, the drying treatment at 45 °C for 18 h significantly reduced the relative humidity (RH) content of the samples from approximately 75% to below 8%. This value is considered safe from a microbiological and an enzymatic point of view. After drying treatment, RH values ranged from 7.89 ± 0.14 to 8.20 ± 0.38% among the dried batch samples.

### 3.2. Volatile Characterization

A total of seventy-six volatile compounds were identified and categorized into various chemical families. These included twenty-three monoterpenes that were hydrocarbonated, three monoterpenes that were oxygenated, twenty-five sesquiterpenes that were hydrocarbonated, three alcohols, twelve aldehydes, three ketones, three esters, two furanic compounds and two C-13 norisoprenoids. It is important to note that not all of these compounds were present in every sample.

#### 3.2.1. Monoterpenes

Volatile terpenes represent the main group of odor-active volatile components of the mango’s overall volatilome, exhibiting green, herbaceous, citrus, resinous and woody scents [17]. Within this mango peel volatile profile, two chemical groups of monoterpenes can be found: hydrocarbonated and oxygenated ones. Concerning hydrocarbonated monoterpenes, it can be observed that fresh samples, regardless of their maturation stage, exhibit a significantly higher total concentration of these compounds compared to dried samples (Table 2). When observing the differences among the fresh samples, it is clear that the peel sample from the optimum ripening stage showed the highest concentration of hydrocarbonated terpenes (135.60 ± 27.19 μg/g DM), which differed statistically from both the fresh green and the fresh overripe samples (98.75 ± 7.37 and 73.49 ± 13.40 μg/g DM, respectively). Among these terpenes, 3-carene was the predominant compound. The highest concentration of 3-carene was found in the fresh optimum sample, accounting for 102.37 ± 20.40 μg/g DM, followed by fresh green and fresh overripe samples exhibiting total values of 72.36 ± 6.28 μg/g DM and 56.67 ± 13.65 μg/g DM, respectively. A substantial loss of hydrocarbonated monoterpenes was observed after drying, resulting in total contents of 30–35 μg/g DM. Regardless of this reduction, 3-carene consistently dominated the profile, with values close to 25 μg/g DM in all dried samples. This trend also appears in other compounds belonging to this family, such as α-phellandrene and δ-limonene, being indeed the ones that contributed the most to the overall terpenic mango aroma due to their low odor thresholds [8]. The literature has observed a reduction in the content of monoterpenes when vegetable matrices are subjected to heating procedures. This reduction is particularly evident in the case of mango fruit [17,30] as well as in the case of other fruits, spices and vegetables [31,32,33,34].

In the case of the oxygenated terpenes (Table 2) group, the highest total content of these compounds was found in the fresh optimum sample, with linalool being the primary contributor. Minor amounts of eucarvone and citral were detected in the samples. After drying, linalool levels markedly decreased and no significant differences were observed among maturity stages in the dried samples. In contrast, thermal processing has been reported to promote the release of specific compounds within this group [19,35], as observed for citral. Higher citral contents were detected in all dried samples, independent of maturity stage, reaching 0.04 ± 0.00 μg/g DM in dried green samples and 0.02 ± 0.00 μg/g DM in both dried ripe and overripe samples.

#### 3.2.2. Sesquiterpenes

Sesquiterpenes, another big group of organic compounds that plays a crucial role in contributing to the overall volatile composition of mango [10,20,29], showed notable variations in quantity between fresh and dried mango peel samples (Table 3). Differences among ripening stages were more evident in fresh samples than those observed for monoterpenes, as statistically significant variations were detected across all three maturity stages. The fresh sample at the optimal ripening stage showed the highest total concentration (47.10 ± 10.83 μg/g DM), whereas lower values were disclosed for green (18.75 ± 1.12 μg/g DM) and overripe samples (22.84 ± 1.15 μg/g DM). This profile was mainly driven by *β*-selinene, *trans-β*-caryophyllene and *α*-humulene. A similar pattern was observed after drying, although overall concentrations decreased by approximately two- to three-fold compared to fresh samples. Among dried samples, the optimally ripe peel exhibited the highest content (15.51 ± 4.17 μg/g DM), while no significant differences were found between the least and most ripe stages, with values of 7.50 ± 1.92 and 8.15 ± 1.16 μg/g DM, respectively. In all cases, a similar trend observed for monoterpenes was found: heat treatment promotes the compounds to volatilize and escape from the matrix as lower concentrations of these compounds are found in the heat-treated samples. These results are aligned with the outcomes observed in the literature when studying similar matrices and treatments [33,36].

These differences appeared to be due to the fact that several sesquiterpenes are scarcely present in the samples at an earlier stage of maturation. These compounds are likely formed and developed as the maturation process progresses, reaching their peak during optimal ripening and gradually decreasing throughout the ripening stage [20,37].

#### 3.2.3. Alcohols, Aldehydes, Ketones and Esters

Other families composing the mango volatilome, such as alcohols and esters (Table 4), were identified in lower quantitative proportions compared to monoterpenes and sesquiterpenes. These compounds, in conjunction with aldehydes and ketones, play a pivotal role in shaping the sensory profile of fruits by contributing characteristic fatty-grassy and green-fruity aromas [38].

The highest levels were observed in fresh overripe samples, reaching 0.09 ± 0.01 μg/g DM for alcohols and 0.07 ± 0.01 μg/g DM for esters. These findings are consistent with previous reports indicating that compounds belonging to these classes progressively accumulate throughout the ripening process [39]. However, when examining their differences in terms of ripeness stage and drying treatment, they exhibited similar behaviors: dried samples demonstrated no statistically significant differences, irrespective of the ripening stage, with values around 0.05 μg/g DM for alcohols and 0.02 μg/g DM for esters.

Regarding aldehydes and ketones, it could be seen that the highest total quantity of aldehydes was found in the fresh sample at its optimum ripening stage, accounting for 0.77 ± 0.11 μg/g DM. (Table 4). Meanwhile, the highest concentration of ketones was detected in the fresh green sample (43.42 ± 3.06 μg/g DM). The drying process led to a pronounced decrease in the levels of these compounds. This effect was especially marked for ketones, whose total content was reduced by nearly 85% in the samples and by approximately 90% in overripe samples. In comparison, aldehydes showed a lower sensitivity to thermal treatment, with losses of around 50% in both maturity stages. By contrast, this drying-related reduction was not observed in immature peel samples, as comparable concentrations were found between fresh and dried green mango peel.

#### 3.2.4. Furanic Compounds and C13 Norisoprenoids

With respect to furanic compounds, the most notable change was the absence of furfural in fresh mango peel, followed by its appearance after drying (Table 5). This fact seemed to be attributable to Maillard and caramelization reactions in which carbohydrates subjected to extreme temperatures are susceptible to being transformed into derived compounds, such as furfural. This behavior has been observed in similar vegetable matrices [40,41]. Within these reactions, the less polymeric the carbohydrate, the more sensitive to undergo those transformations [24]. Accordingly, the dried overripe sample exhibited the highest furfural concentration, which can be linked to its higher simple carbohydrate content resulting from enhanced enzymatic saccharification during fruit ripening [12]. In this sample, furfural reached 14.47 ± 2.21 ng/g DM, a significantly higher value compared to the other dried samples, which showed concentrations of 3.09 ± 0.33 and 2.31 ± 0.67 ng/g DM. This occurrence may provide the scent of this sample with sweet caramel and toasty sensory notes.

*β*-damascenone and *β*-ionone (Table 5) are known for their distinct rose- and violet-like scents, respectively. Due to their low detection thresholds, these compounds have been proposed as key odorants contributing to the overall aroma profile of several mango cultivars [10,13,29]. These two compounds were present in all the dried samples except for the fresh green one. This absence might be attributed to the inherent synthesis process of these compounds, which likely has not yet fully developed at this stage of the fruit maturity, as happens in other fruit matrices [42]. For the remaining fresh samples (both ripe and overripe), while these compounds were detected in the chromatograms, their concentrations were too low to allow for accurate quantification. In contrast, significantly higher amounts of these compounds were found in the samples that had undergone heat treatment. The highest concentrations of *β*-damascenone and *β*-ionone were observed in the dried samples at the optimal ripening stage, accounting for 25.21 ± 5.48 ng/g DM and 33.44 ± 6.64 ng/g DM, respectively. As happens with the occurrence of the furanic compounds, the quantities of some C13-norisoprenoids, such as *β*-damascenone and *β*-ionone, are augmented when subjecting them to heat conditions. However, in this case, this enhancement occurs due to the release of the free volatile aglycones as a consequence of the saccharification hydrolysis reactions induced by thermal processes [25,43].

### 3.3. Hierarchical Clustered Heatmap

**Figure 1:**
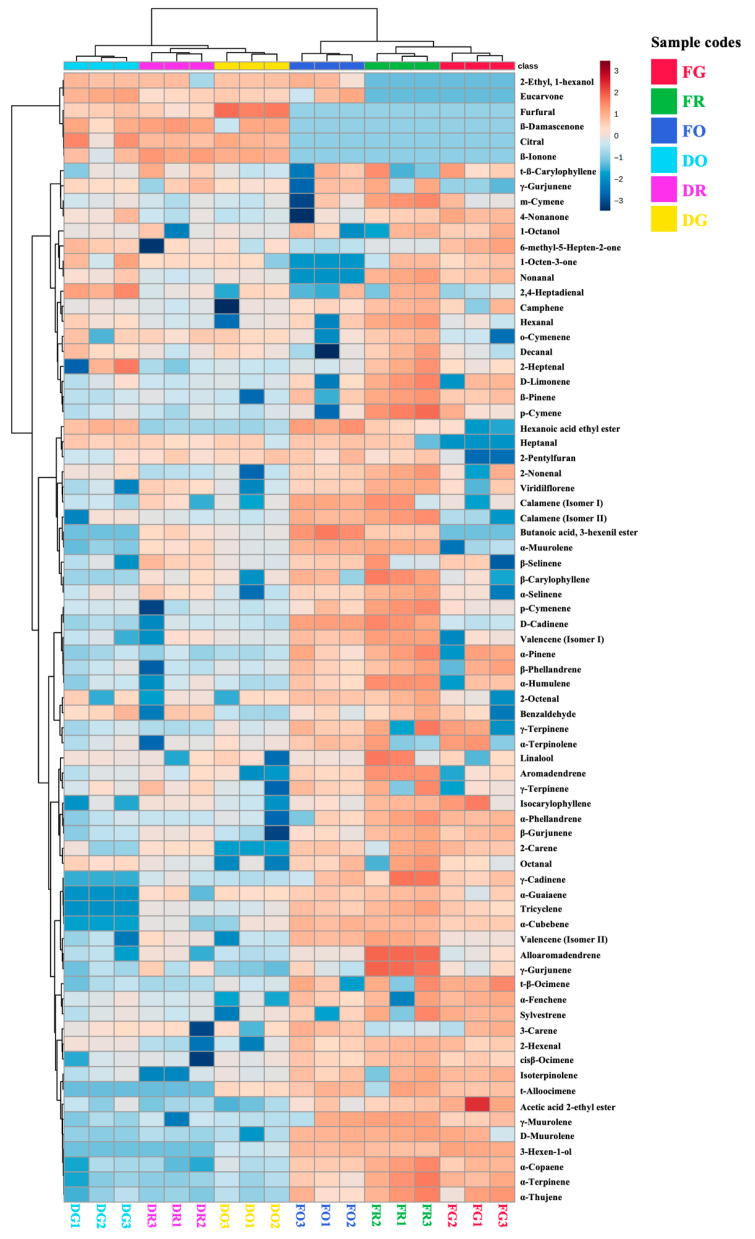
Hierarchical clustered heatmap graph of fresh and dried mango peel samples at three different ripening stages. “FG”, fresh green; “FR”, fresh ripe; “FO”, fresh overripe; “DG”, dried green; “DR” dried ripe; “DO” dried overripe. Euclidean measure distance and Ward’s linkage were considered when performing the agglomerative hierarchical clustering.

To identify the volatile compounds most responsible for differentiating fresh and dried samples across ripening stages, a hierarchical clustering heatmap with associated dendrograms was generated (Figure 1). The heatmap analysis, performed using MetaboAnalyst 5.0, provides a clear visualization of how the volatile composition of mango peels varies with ripening stage and drying treatment. The dataset was log-transformed, auto-scaled and clustered using Euclidean distance and Ward’s linkage to group samples with similar volatilome profiles. Each column represents a sample replicate, and each row corresponds to an individual compound, with color intensity (red to blue) indicating relative abundance.

The hierarchical clustering, represented by the dendrogram, clearly distinguishes two major groups: fresh samples and dried samples. A dotted black line separates these groups, indicating substantial differences in volatile composition due to the drying treatment. The fresh samples, located on the right side of the heatmap, exhibit more pronounced color variations across the columns, suggesting greater variability in volatile composition among samples at different maturity stages. This indicates that the maturity stage significantly affects the volatilome profile when analyzing fresh samples.

In contrast, the dried samples, positioned on the left side of the heatmap, show less variability in the range of color, which implies that the drying process dampens some of the differences found among the fresh samples, thus homogenizing the volatile composition among the dried samples. This reduced variability is also reflected in the dendrogram clustering, where the dried samples form tighter, more cohesive clusters compared to the fresh samples. While the maturity stage continues to be an influence on the volatile composition of dried samples, its impact is notably less pronounced compared to the influences exhibited among the fresh samples. This observation suggests that the drying treatment, unavoidable for revalorization purposes, minimizes the differences among the ripening stages: mango peel by-products from green, ripe and overripe fruits are considered equally suitable for upcycling. This fact helps to broad the circularity and sustainability associated with mango processing industries.

### 3.4. Principal Component Analysis

**Figure 2:**
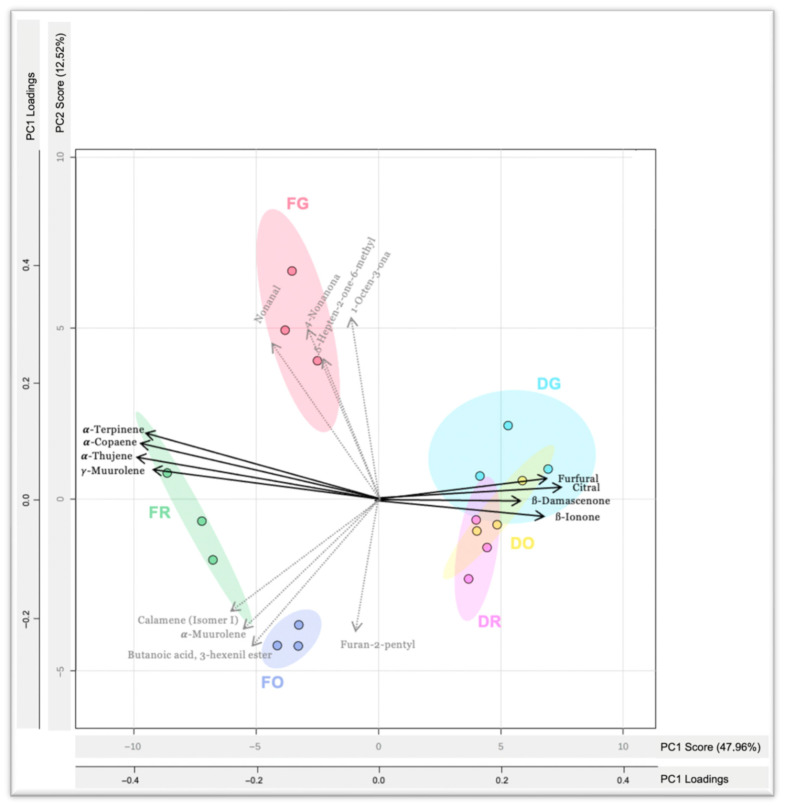
PCA score and loading biplot illustrating the distribution of fresh and dried mango peel samples across three ripening stages based on PC1 and PC2. Sample codes are as follows: FG, fresh green; FR, fresh ripe; FO, fresh overripe; DG, dried green; DR, dried ripe; DO, dried overripe. PC1 loadings are indicated by black arrows, whereas PC2 loadings are represented by grey dotted arrows.

To further investigate the volatile compounds contributing most to sample differentiation, principal component analysis (PCA) was applied, including a biplot displaying the four variables with the highest contribution to variance in each component (Figure 2). This analysis helps visualize how ripening and drying jointly shape the volatile composition of mango peels.

The first two principal components explained a 60.48% of the variance between all the samples. Principal component 1 (PC1) clearly separates the fresh samples from the dried ones, highlighting the dominant effect of drying on the volatilome. Some hydrocarbonated terpenes (*α*-terpinene, *α*-copaene, *α*-thujene and ɣ-muurolene) were correlated with the fresh samples (negative axis of PC1). This representation is explained by the high content of hydrocarbonated monoterpenes of fresh samples, which ranged from 74 to 135 μg/g DM in comparison with that of dried ones, whose contents were heavily decreased (30 to 35 μg/g DM). In contrast, dried samples were associated with *β*-damascenone and *β*-ionone (C13-norisoprenoids), citral (oxygenated monoterpene) and furfural (furanic compound), which loaded positively on PC1. As discussed previously, these compounds were generally absent in fresh samples, as they are mainly formed or released as a consequence of thermal processing.

Principal Component 2 accounted for 12.52% of the total variance and enabled discrimination among samples according to ripening stage. Along this component, less mature samples (green fresh and dried) were positioned on the positive side, while samples at optimal ripeness and overripe stages, regardless of processing, were located on the negative side. This separation was mainly driven by several ketones, including 4-nonanone, 5-hepten-2-one-6-methyl, nonanal and 1-octen-3-one, which were associated with the green samples. In contrast, ripe and overripe samples correlated with a set of volatiles from different aroma classes, such as α-muurolene, calamene (isomer 1) and butanoic acid, 3-hexenyl ester. This separation reflects the influence of ripening stage on specific compound families, where greener samples are characterized by ketones and aldehydes, whilst riper ones show a higher presence of esters and terpenes. If considering PC3, with an additional 10.13% accumulated variance, this increases up to 70.61%, providing additional structure in the maturity stage sample differentiation, with aldehydes like decanal, 2,4-heptadienal and nonanal emerging as key contributors in the positive axis of this PC. Meanwhile, terpenes as 3-carene, α-terpinolene and *β*-selinene were among the most influential volatiles on the negative side of PC3.

### 3.5. Sensory Evaluation

The olfactive evaluation revealed sensory differences among mango peel samples at different ripening stages and processing conditions (Table 6). The fresh green sample showed pronounced herbaceous, pine-like and resinous notes, typical of unripe mango tissues, while the ripe sample presented the most balanced expression, with the higher citrus and pine intensities characteristic of optimal maturity [21]. In contrast, fresh overripe peel stood out for its distinctly higher floral intensity compared with FG and FR (differences confirmed by the SNK test), reflecting the loss of fresh-green volatiles and the evolution towards the more floral, ripe-fruit aromas commonly associated with late maturation [12].

The thermal processing applied, drying, modified all these profiles across all ripening stages. The three different dried samples exhibited lower intensities of herbaceous, citrus and pine descriptors but showed a consistent presence of floral and caramel/honey-like notes (no significant differences among DG, DR and DO were found for these attributes). These occurrences were most pronounced in dried ripe and overripe samples, likely linked to a boosted thermally driven formation of Maillard compounds. This behavior, linked to the reduction in the original fresh profiles, contributed to uniform sweet and floral aromas among all the dried samples [36,44].

The sensory evaluation revealed that none of the dried samples exhibited off-flavors, and all ripening stages were consistently perceived as having a pleasant and acceptable aromatic profile, with no significant differences in overall olfactory quality among them.

**Table 6:** Olfactive sensory evaluation using a 10 cm unstructured scale of fresh and dried mango peel samples (*n* = 8).

	Fresh Mango Peels			Dried Mango Peels		
	Green	Ripe	Overripe	Green	Ripe	Overripe
Olfactive Descriptor						
Green/Herbaceous	7.9 ± 1.1 ^a^	6.6 ± 0.3 ^b^	4.8 ± 0.3 ^c^	3.3 ± 0.8 ^c^	4.3 ± 2.3 ^c^	3.9 ± 1.2 ^c^
Citrus	6.9 ± 0.9	7.8 ± 1.3	5.3 ± 2.1	6.2 ± 0.6	6.5 ± 0.2	5.8 ± 1.1
Pine/Resinous	7.0 ± 0.2 ^b^	8.9 ± 1.2 ^a^	6.5 ± 1.1 ^b^	3.3 ± 1.8 ^c^	4.0 ± 2.1 ^c^	3.0 ± 0.2 ^c^
Floral	2.4 ± 0.9 ^c^	6.3 ± 0.5 ^b^	8.5 ± 0.3 ^a^	7.4 ± 1.1 ^a^	8.3 ± 1.4 ^a^	8.0 ± 1.0 ^a^
Caramel/Honey-like	ND	ND	ND	7.5 ± 0.8	8.0 ± 1.6	8.6 ± 1.2

Values with different superscripts in the same row denote significant differences according to the Student–Newman–Keuls test at *p* ˂ 0.05. ND: Non-detected.

## 4. Conclusions

Findings: Ripening stage significantly influences the volatile profile of fresh mango peels. The highest overall volatile content across nearly all compound classes was observed in fresh peels at the optimal ripening stage. However, thermal drying treatments (unavoidable steps for subsequent valorization applications) equalize volatile profiles across maturity stages, dampening the differences attributable to ripening. These findings highlight the potential for mango side-streams to be uniformly stabilized and upcycled as aroma-rich ingredients supporting industrial decision-making in circular food systems.

Research limitations: The research was limited to three specific ripening stages and a single mango species, so other factors were not explored and may influence volatile profiles. Additionally, the practical revalorization of stabilized peels was not assessed in this study.

Future research should focus on evaluating both the feasibility and practical revalorization applicability of these side-streams. Such efforts are considered essential to apply these findings to real-world applications and to fully contribute to value-driven food waste management strategies.

## Figures and Tables

**Table 1:** Relative humidity content, expressed in percentage, (*n* = 3) of the total six mango peel samples at different treatment conditions (fresh or oven-dried at 45 °C for 18 h) and maturity stages (green, ripe and overripe).

Sample	Maturity Stage	Relative Humidity (%)
Fresh Mango Peels	Green	78.16 ± 0.63 ^c^
	Ripe	75.38 ± 0.77 ^b^
	Overripe	74.25 ± 0.27 ^b^
Oven-dried Mango Peels	Green	7.89 ± 0.14 ^a^
	Ripe	8.20 ± 0.38 ^a^
	Overripe	8.06 ± 0.04 ^a^

Values with different superscripts in the same column denote significant differences according to the Student–Newman–Keuls test at *p* ˂ 0.05.

**Table 2:** Monoterpenes content (μg/g DM, mean ± SD, *n* = 3) in fresh and dried mango peel at different ripening stages.

	Fresh Mango Peels			Dried Mango Peels		
Volatile Compounds	Green	Ripe	Overripe	Green	Ripe	Overripe
*α*-Pinene	0.85 ± 0.1 ^b^	0.97 ± 0.21 ^b,c^	0.61 ± 0.25 ^b^	0.20 ± 0.05 ^a^	0.20 ± 0.05 ^a^	0.21 ± 0.02 ^a^
*α*-Fenchene	0.08 ± 0.01 ^b,c^	0.09 ± 0.02 ^b,c^	0.05 ± 0.02 ^b^	0.02 ± 0.00 ^a^	0.01 ± 0.00 ^a^	0.02 ± 0.00 ^a^
Camphene	0.02 ± 0.01 ^a,b^	0.02 ± 0.00 ^b^	0.01 ± 0.00 ^a^	0.01 ± 0.00 ^a^	0.01 ± 0.00 ^a^	0.01 ± 0.00 ^a^
Tryciclene	0.01 ± 0.00	0.02 ± 0.00	0.02 ± 0.00	Tr	0.01 ± 0.00	0.01 ± 0.00
*β*-Pinene	0.03 ± 0.05 ^b,c^	0.06 ± 0.01 ^b,c^	0.03 ± 0.00 ^b^	0.01 ± 0.00 ^a^	0.01 ± 0.00 ^a^	0.01 ± 0.00 ^a^
*2*-Carene	0.17 ± 0.05 ^b^	0.31 ± 0.06 ^c^	0.14 ± 0.03 ^b^	0.07 ± 0.02 ^a^	0.07 ± 0.01 ^a^	Tr
*3*-Carene	72.36 ± 6.28 ^b,c^	102.37 ± 20.40 ^b,c^	56.67 ± 13.65 ^b^	22.73 ± 0.93 ^a^	25.58 ± 2.49 ^a^	26.67 ± 1.96 ^a^
α-Phellandrene	4.52 ± 0.27 ^b^	5.95 ± 1.25 ^b^	2.37 ± 1.47 ^a^	1.19 ± 0.39 ^a^	1.34 ± 0.01 ^a^	1.66 ± 0.17 ^a^
*α*-Terpinene	1.17 ± 0.11 ^c,d^	1.54 ± 0.31 ^d^	0.81 ± 0.14 ^c^	0.29 ± 0.08 ^a^	0.33 ± 0.14 ^a,b^	0.40 ± 0.05 ^b^
Silvestrene	0.39 ± 0.04	0.55 ± 0.10 ^d^	0.27 ± 0.02 ^b^	0.12 ± 0.03 ^a^	0.12 ± 0.03 ^a^	0.16 ± 0.02 ^a^
*δ*-Limonene	4.29 ± 0.23 ^c^	5.79 ± 1.14 ^c^	3.00 ± 0.23 ^b^	1.85 ± 0.59 ^a^	1.56 ± 0.09 ^a^	1.67 ± 0.17 ^a^
*β*-Phellandrene	1.97 ± 0.18 ^d^	1.77 ± 0.39 ^d^	1.22 ± 0.28 ^c^	0.47 ± 0.12 ^a^	0.43 ± 0.00 ^a^	0.55 ± 0.05 ^b^
*cis-β*-Ocymene	0.04 ± 0.01 ^b^	0.07 ± 0.01 ^d^	0.04 ± 0.01 ^b^	0.01 ± 0.00 ^a^	0.01 ± 0.00 ^a^	0.02 ± 0.00 ^a^
*α*-Thujene	0.09 ± 0.03 ^b,c^	0.11 ± 0.02 ^c^	0.07 ± 0.02 ^b^	0.03 ± 0.01 ^a^	0.03 ± 0.00 ^a^	0.03 ± 0.00 ^a^
*γ*-Terpinene	0.25 ± 0.01 ^d^	0.30 ± 0.06 ^d^	0.18 ± 0.03 ^c^	0.07 ± 0.01 ^a^	0.07 ± 0.00 ^a^	0.10 ±0.01 ^b^
*trans-β*-Ocymene	0.31 ± 0.08 ^c^	0.33 ± 0.06 ^c^	0.20 ± 0.06 ^b^	0.05 ± 0.02 ^a^	0.05 ± 0.00 ^a^	0.07 ±0.01 ^a^
*m*-Cymene	0.11 ± 0.05 ^b^	0.24 ± 0.03 ^c^	0.10 ± 0.04 ^b^	0.08 ± 0.01 ^a^	0.06 ± 0.01 ^a^	0.07 ± 0.01 ^a^
*p*-Cymene	0.93 ± 0.34 ^b^	1.92 ± 0.30 ^c^	0.85 ± 0.28 ^b^	0.52 ± 0.09 ^a^	0.49 ± 0.09 ^a^	0.52 ±0.07 ^a^
*iso*-Terpinolene	1.26 ± 0.13 ^b,c^	1.43 ± 0.30 ^c^	0.98 ± 0.20 ^b^	0.30 ± 0.08 ^a^	0.33 ± 0.01 ^a^	0.39 ± 005 ^a^
*α*-Terpinolene	9.52 ± 1.36	10.69 ± 2.40	5.33 ± 0.53	2.15 ± 0.58	2.48 ± 0.09	2.96 ± 0.49
*trans*-Allocymene	0.02 ± 0.00 ^a^	0.05 ± 0.01 ^b^	0.02 ± 0.01 ^a^	ND	ND	0.01 ± 0.00 ^a^
*o*-Cymenene	0.03 ± 0.00 ^a^	0.11 ± 0.02 ^b^	0.06 ± 0.00 ^a^	0.10 ± 0.02 ^b^	0.08 ± 0.02 ^b^	0.08 ± 0.01 ^b^
*p*-Cymenene	0.32 ± 0.03 ^b^	0.91 ± 0.10 ^c^	0.46 ± 0.13 ^b^	0.23 ± 0.01 ^a^	0.21 ± 0.04 ^a^	0.22 ± 0.02 ^a^
***Σ Hydrocarbonated monoterpenes***	**98.75 ± 7.37 ^b^**	**135.60 ± 27.19 ^c^**	**73.49 ± 13.40 ^b^**	**30.49 ± 7.99 ^a^**	**33.49 ± 2.30 ^a^**	**35.82 ± 2.78 ^a^**
Linalool	0.01 ± 0.00 ^a^	0.09 ± 0.02 ^b^	0.01 ± 0.00 ^a^	0.01 ± 0.00 ^a^	0.01 ± 0.00 ^a^	0.02 ± 0.00 ^a^
Eucarvone	Tr	Tr	Tr	Tr	Tr	Tr
Citral	Tr	Tr	Tr	0.04 ± 0.00 ^b^	0.02 ± 0.00 ^a^	0.02 ± 0.00 ^a^
***Σ Oxigenated monoterpenes***	**0.01 ± 0.00 ^a^**	**0.09 ± 0.02 ^d^**	**0.01 ± 0.00 ^a^**	**0.06 ± 0.00 ^b,^ ^c^**	**0.03 ± 0.00 ^b^**	**0.04 ± 0.01 ^b^**

Different superscript letters within the same row indicate statistically significant differences (Student–Newman–Keuls test, *p* < 0.05). ND, not detected; Tr, traces.

**Table 3:** Sesquiterpenes content (μg/g DM, mean ± SD, *n* = 3) in fresh and dried mango peel at different ripening stages.

	Fresh Mango Peels			Dried Mango Peels		
Volatile Compounds	Green	Ripe	Overripe	Green	Ripe	Overripe
*α*-Cubebene	0.10 ± 0.01 ^b^	0.19 ± 0.00 ^c^	0.21 ± 0.04 ^c^	Tr	0.03 ± 0.00 ^a^	0.04 ± 0.00 ^a^
*α*-Copaene	2.13 ± 0.31 ^c^	2.97 ± 0.52 ^d^	1.84 ± 0.02 ^c^	0.58 ± 0.21 ^a^	0.49 ± 0.16 ^a^	0.83 ± 0.14 ^b^
*α*-Gurjunene	0.14 ±0.05 ^a^	2.35 ± 0.04 ^d^	1.50 ± 0.06 ^c^	0.88 ± 0.06 ^b^	1.56 ± 0.36 ^c^	0.77 ± 0.09 ^b^
*β-G*urjunene	0.12 ± 0.02 ^c^	0.14 ± 0.02 ^c^	0.07 ±0.01 ^b^	0.03 ± 0.01 ^a^	0.06 ± 0.00 ^b^	0.03 ± 0.00 ^a^
*α*-Guaiaene	0.02 ± 0.00	0.03 ± 0.01	0.02 ± 0.00	0.00 ± 0.02	0.01 ± 0.00	0.01 ± 0.00
*β*-Caryophyllene	0.05 ± 0.01 ^b^	0.25 ± 0.06 ^d^	0.14 ± 0.01 ^c^	0.02 ± 0.00 ^a^	0.05 ± 0.01 ^b^	0.05 ± 0.01 ^b^
*trans-β*-Caryophyllene	4.78 ± 1.68 ^b^	10.24 ±2.08 ^d^	5.12 ± 0.65 ^c^	2.31 ± 0.79 ^a^	4.28 ± 1.50 ^b^	2.30 ± 0.34 ^a^
Aromadendrene	0.16 ± 0.03 ^b^	0.59 ± 0.00 ^c^	0.20 ± 0.02 ^b^	0.08 ± 0.02 ^a^	0.12 ± 0.05 ^a,b^	0.12 ± 0.02 ^a,b^
Alloaromadendrene	0.05 ± 0.02 ^b^	0.68 ± 0.03 ^c^	0.05 ± 0.00 ^b^	0.02 ± 0.01 ^a^	0.05 ± 0.00 ^b^	0.02 ± 0.00 ^a^
*iso*-Caryophyllene	0.16 ± 0.04 ^c^	0.06 ± 0.00 ^b^	0.02 ± 0.00 ^a^	0.01 ± 0.00 ^a^	0.02 ± 0.00 ^a^	0.01 ± 0.00 ^a^
*γ*-Gurjunene	0.12 ± 0.02 ^b^	0.34 ± 0.02 ^c^	0.11 ± 0.04 ^b^	0.06 ± 0.01 ^a^	0.12 ± 0.04 ^b^	0.05 ± 0.01 ^a^
*α*-Humulene	4.39 ± 0.33 ^c^	8.47 ± 0.35 ^d^	3.75 ± 0.62 ^b,c^	1.39 ± 0.43 ^a^	2.47 ± 0.85 ^a,b^	1.35 ± 0.22 ^a^
*γ*-Selinene	0.18 ± 0.06 ^b^	0.46 ± 0.10 ^d^	0.24 ± 0.05 ^c^	0.13 ± 0.04 ^a,b^	0.22 ± 0.08 ^c^	0.10 ± 0.01 ^a^
*α*-Muurolene	0.08 ± 0.01 ^a^	0.82 ± 0.04 ^d^	0.75 ± 0.04 ^d^	0.05 ± 0.01 ^a^	0.32 ± 0.05 ^c^	0.17 ± 0.02 ^b^
*γ*-Muurolene	0.35 ± 0.09 ^b^	0.79 ± 0.01 ^d^	0.68 ± 0.05 ^c^	0.05 ± 0.01 ^a^	0.08 ± 0.02 ^a^	0.07 ± 0.01 ^a^
Viridiflorene	0.34 ± 0.06 ^b^	0.72 ± 0.04 ^d^	0.37 ± 0.02 ^b^	0.11 ± 0.03 ^a^	0.34 ± 0.06 ^b^	0.14 ± 0.02 ^a^
Valencene (*isomer i*)	0.15 ± 0.00 ^b^	0.58 ± 0.08 ^d^	0.43 ± 0.02 ^c^	0.06 ± 0.01 ^a^	0.18 ± 0.05 ^b^	0.08 ± 0.01 ^a^
Valencene (*isomer ii*)	0.10 ± 0.02 ^b^	0.64 ± 0.04 ^d^	0.27 ± 0.04 ^c^	0.04 ± 0.01 ^a^	0.12 ± 0.03 ^b^	0.05 ± 0.01 ^a^
*β*-Selinene	3.55 ± 0.08 ^b^	10.59 ± 1.20 ^d^	3.50 ± 0.23 ^b^	1.06 ± 0.29 ^a^	3.82 ± 1.05 ^b^	1.35 ± 0.20 ^a^
*α*-Selinene	0.49 ± 0.12 ^a^	1.55 ± 0.22 ^d^	0.78 ± 0.02 ^b^	0.39 ± 0.10 ^a^	0.75 ± 0.16 ^b^	0.29 ± 0.04 ^a^
*δ*-cadinene	0.23 ± 0.02 ^b^	2.09 ± 0.51 ^c^	1.80 ± 0.02 ^c^	0.16 ± 0.03 ^a^	0.27 ± 0.03 ^b^	0.23 ± 0.04 ^b^
*γ*-Cadinene	0.26 ± 0.06 ^c^	1.66 ± 0.09 ^e^	0.41 ± 0.07 ^d^	Tr	0.06 ± 0.02 ^b^	0.03 ± 0.00 ^a^
*δ-M*uurolene	0.27 ± 0.03 ^b,c^	0.30 ± 0.05 ^c^	0.23 ± 0.02 ^b^	0.01 ± 0.00 ^a^	0.01 ± 0.00 ^a^	0.02 ± 0.00 ^a^
Calamene *(Isomer I)*	0.01 ± 0.00 ^a^	0.31 ± 0.08 ^c^	0.16 ± 0.03 ^b^	0.04 ± 0.01 ^a^	0.02 ± 0.01 ^a^	0.02 ± 0.00 ^a^
Calamene *(Isomer II)*	0.04 ± 0.00 ^a^	0.25 ± 0.01 ^d^	0.18 ± 0.02 ^c^	0.02 ± 0.01 ^a^	0.06 ± 0.02 ^b^	0.03 ± 0.00 ^a^
***Σ Sesquiterpenes***	**18.25 ± 1.12 ^b^**	**47.10 ± 3.83 ^d^**	**22.84 ± 1.15 ^c^**	**7.50 ± 1.92 ^a^**	**15.51 ± 4.17 ^b^**	**8.15 ± 1.16 ^a^**

Different superscript letters within the same row indicate statistically significant differences (Student–Newman–Keuls test, *p* < 0.05). Tr, traces.

**Table 4:** Alcohols, aldehydes, esters content (μg/g DM, mean ± SD, *n* = 3) in fresh and dried mango peel at different ripening stages.

	Fresh Mango Peels			Dried Mango Peels		
Volatile Compounds	Green	Ripe	Overripe	Green	Ripe	Overripe
(Z)-3-Hexen-1-ol	0.04 ± 0.00 ^b^	0.02 ± 0.00 ^a^	0.02 ± 0.00 ^a^	Tr	Tr	Tr
2-Ethyl, 1-Hexanol	Tr	Tr	0.05 ± 0.01 ^b^	0.04 ± 0.01 ^a^	0.03 ± 0.00 ^a^	0.03 ± 0.00 ^a^
1-Octanol	0.02 ± 0.01 ^a,b^	0.03 ± 0.00 ^b^	0.02 ± 0.01 ^a,b^	0.01 ± 0.00 ^a^	0.02 ± 0.01 ^a,b^	0.01 ± 0.00 ^a^
***Σ Alcohols***	**0.06 ± 0.00 ^b^**	**0.05 ± 0.00 ^a^**	**0.09 ± 0.01 ^c^**	**0.05 ± 0.01 ^a^**	**0.05 ± 0.00 ^a^**	**0.04 ± 0.00 ^a^**
Pentanal	0.02 ± 0.01	0.02 ± 0.00	0.01 ± 0.00	0.02 ± 0.00	0.01 ± 0.00	0.01 ± 0.00
Hexanal	0.04 ± 0.01 ^a^	0.13 ±0.01 ^c^	0.07 ± 0.01 ^b^	0.06 ± 0.01 ^b^	0.03 ± 0.00 ^a^	0.04 ± 0.00 ^a^
Heptanal	Tr	0.05 ± 0.00 ^a,b^	0.05 ± 0.00 ^a,b^	0.04 ± 0.01 ^a^	0.03 ± 0.01 ^a^	0.04 ± 0.00 ^a^
2-Hexenal	0.12 ± 0.03 ^c^	0.16 ± 0.02 ^c^	0.11 ± 0.03 ^c^	0.06 ± 0.00 ^b^	0.03 ± 0.00 ^a^	0.04 ± 0.01 ^a^
Octanal	0.03 ± 0.01 ^a^	0.06 ± 0.01 ^b^	0.04 ± 0.01 ^a,b^	0.03 ± 0.00 ^a^	0.02 ± 0.00 ^a^	0.03 ± 0.00 ^a^
2-Heptenal	0.01 ± 0.00	0.01 ± 0.00	0.01 ± 0.00	0.01 ± 0.00	ND	ND
Nonanal	0.07 ± 0.01 ^b^	0.10 ± 0.00 ^c^	0.07 ± 0.00 ^b^	0.05 ± 0.01 ^a^	0.03 ± 0.00 ^a^	0.04 ± 0.00 ^a^
2-Octenal-(E)	0.02 ± 0.00 ^a^	0.05 ± 0.02 ^a,b^	0.04 ± 0.00 ^a,b^	0.03 ± 0.00 ^a^	0.02 ± 0.00 ^a^	0.03 ± 0.00 ^a^
(2E, 4E)-Hepta-2,4-dienal	ND	0.02 ± 0.00	0.01 ± 0.00	0.02 ± 0.01	0.01 ± 0.00	0.01 ± 0.00
Decanal	0.07 ± 0.01 ^a^	0.13 ± 0.03 ^b^	0.07 ± 0.02 ^a^	0.10 ± 0.02 ^a,b^	0.07 ± 0.01 ^a^	0.09 ± 0.00 ^a^
Benzaldehyde	0.01 ± 0.00	0.01 ± 0.00	0.01 ± 0.00	0.01 ± 0.00	0.01 ± 0.00	Tr
2-Nonenal	0.02 ± 0.01 ^a^	0.03 ± 0.00 ^a,b^	0.01 ± 0.00 ^a^	0.01 ± 0.00 ^a^	0.01 ± 0.00 ^a^	0.01 ± 0.00 ^a^
***Σ Aldehydes***	**0.40 ± 0.04 ^c^**	**0.77 ± 0.11 ^d^**	**0.50 ± 0.02 ^c^**	**0.45 ± 0.06 ^c^**	**0.27 ± 0.02 ^a^**	**0.34 ± 0.00 ^b^**
1-Octen-3-one	6.98 ± 0.69 ^b^	9.67 ± 0.03 ^c^	Tr	13.01 ± 3.99 ^c^	4.13 ± 0.24 ^a^	4.19 ± 0.39 ^a^
4-Nonanone	11.64 ± 1.42 ^a^	8.88 ± 0.54 ^b^	6.04 ± 0.58 ^b^	8.13 ± 2.58 ^a^	4.97 ± 1.00 ^b^	4.73 ± 0.48 ^a^
5-Hepten-2-one, 6-methyl	24.79 ± 3.65 ^d^	10.58 ± 0.10 ^b^	7.75 ± 0.49 ^a^	19.62 ± 2.87 ^c,d^	12.80 ± 2.91 ^c^	12.55 ± 3.91 ^c^
***Σ Ketones***	**43.42 ± 3.06 ^d^**	**29.13 ± 0.41 ^c^**	**13.79 ± 0.11 ^a^**	**40.66 ± 4.60 ^d^**	**21.89 ± 2.83 ^b^**	**21.46 ± 4.04 ^b^**
Hexanoic acid ethyl ester	Tr	Tr	0.01 ± 0.00	0.01 ± 0.00	Tr	Tr
Acetic acid, 2 ethyl hexil ester	0.03 ± 0.00 ^c^	0.02 ± 0.00 ^b^	0.02 ± 0.00 ^b^	0.01 ± 0.00 ^a^	0.01 ± 0.00 ^a^	0.01 ± 0.00 ^a^
Butanoic acid, 3-hexenil ester	ND	0.01 ± 0.00 ^a^	0.04 ± 0.01 ^b^	ND	0.01 ± 0.00 ^a^	Tr
***Σ Esters***	**0.03 ± 0.00 ^b^**	**0.03 ± 0.00 ^b^**	**0.07 ± 0.01 ^c^**	**0.02 ± 0.00 ^a^**	**0.02 ± 0.00 ^a^**	**0.01 ± 0.00 ^a^**

Different superscript letters within the same row indicate statistically significant differences (Student–Newman–Keuls test, *p* < 0.05). ND, not detected; Tr, traces.

**Table 5:** Furanics and C13 norisoprenoids content (μg/g DM, mean ± SD, *n* = 3) in fresh and dried mango peel at different ripening stages.

	Fresh Mango Peels			Dried Mango Peels		
Volatile Compounds	Green	Ripe	Overripe	Green	Ripe	Overripe
Furan-2-pentyl	10.28 ± 2.19 ^a,b^	12.64 ± 2.49 ^b^	14.48 ± 3.78 ^b,c^	8.03 ± 2.24 ^a^	11.73 ± 2.33 ^a,b^	13.73 ± 1.57 ^b^
Furfural	ND	ND	ND	3.09 ± 0.53 ^a^	2.31 ± 0.67 ^a^	14.74 ± 2.21 ^b^
***Σ Furanic compounds***	**10.28 ± 2.19 ^a^**	**12.64 ± 2.49 ^a^**	**14.48 ± 3.78 ^a^**	**11.12 ± 2.77 ^a^**	**14.04 ± 1.80 ^a^**	**28.47 ± 2.19 ^b^**
*β*-Damascenone	ND	Tr	Tr	18.13 ± 2.18 ^a^	25.21 ± 5.48 ^b^	18.31 ± 0.51 ^a^
*β*-Ionone	ND	Tr	Tr	14.15 ± 1.12 ^a^	33.44 ± 6.64 ^c^	22.62 ± 1.91 ^b^
***Σ C13 norisoprenoids***	**ND**	Tr	Tr	**32.28 ± 1.76 ^a^**	**58.64 ± 4.13 ^c^**	**40.93 ± 1.52 ^b^**

Different superscript letters within the same row indicate statistically significant differences (Student–Newman–Keuls test, *p* < 0.05). ND, not detected; Tr, traces.

## Data Availability

The original contributions presented in this study are included in the article. Further inquiries can be directed to the corresponding author.
